# A non-proteolytic function of ubiquitin in transcription repression

**DOI:** 10.15698/mic2014.07.159

**Published:** 2014-07-07

**Authors:** Ada Ndoja, Tingting Yao

**Affiliations:** 1 Department of Biochemistry and Molecular Biology, Colorado State University, Fort Collins, CO 80523, USA.

**Keywords:** transcription, ubiquitination, transcription activator, Cdc48, transcription repression, Met4, R-Smads

## Abstract

Regulation of transcription is vitally important for maintaining normal cellular homeostasis and is also the basis for cellular differentiation, morphogenesis and the adaptability of any organism. Transcription activators, which orchestrate time and locus-specific assembly of complex transcription machinery, act as key players in these processes. One way in which these activators are controlled is by the covalent attachment of the conserved protein, ubiquitin (Ub), which can serve as either a proteolytic or non-proteolytic signal. For a subset of the activators, polyubiquitination-dependent degradation of the activator controls its abundance. In these cases transcription activation can require protein synthesis as well as internal or external stimulus. In contrast, other activators have been reported to undergo mono- or oligoubiquitination that does not lead to protein degradation. The mechanisms by which monoubiquitination of transcription activators affect their activities have been poorly understood. In a recent study, we demonstrated that monoubiquitination of some transcription activators can inhibit transcription by recruiting the AAA+ ATPase Cdc48 (also known in metazoan organisms as p97 or valosin-contain protein, VCP), which then extracts the ubiquitinated activator from DNA.

## Monoubiquitination of a model transcription activator modulates its interaction with DNA

It has long been recognized that a subset of transcription activators contain transcription activation domains that overlap with degrons, sequences within the protein that signal polyubiquitination and subsequent degradation by the proteasome. For some activators, such as Myc and Estrogen Receptor α, transcription activation and activator turnover are interdependent. Much of the initial characterization of this phenomenon utilized a model activator LexA-VP16 (LV), which is a fusion of the DNA binding domain from bacterial LexA and the activation domain from Herpes Simplex Virus protein VP16. LV expressed in yeast is polyubiquitinated by the SCF^Met30^ E3 ligase and rapidly degraded, and this process is coupled to transcription activation of genes placed behind a *LexA* operator. To study the mechanism by which monoubiquitination of activators regulates transcription, we modified this model system by fusing monoUb to the N-terminus of LV. Because wild-type Ub fusions are co-translationally processed by deubiquitinating enzymes (DUBs), we mutated G76 of Ub to V76 to prevent any deubiquitination. To our surprise, the irreversible attachment of monoUb to LV had two effects: (1) it prevented rapid turnover of LV, and (2) Ub-LV no longer activated transcription. These observations prompted us to investigate how monoubiquitination and polyubiquitination of transcription activators can have opposite consequences in transcriptional output.

**Figure 1:**
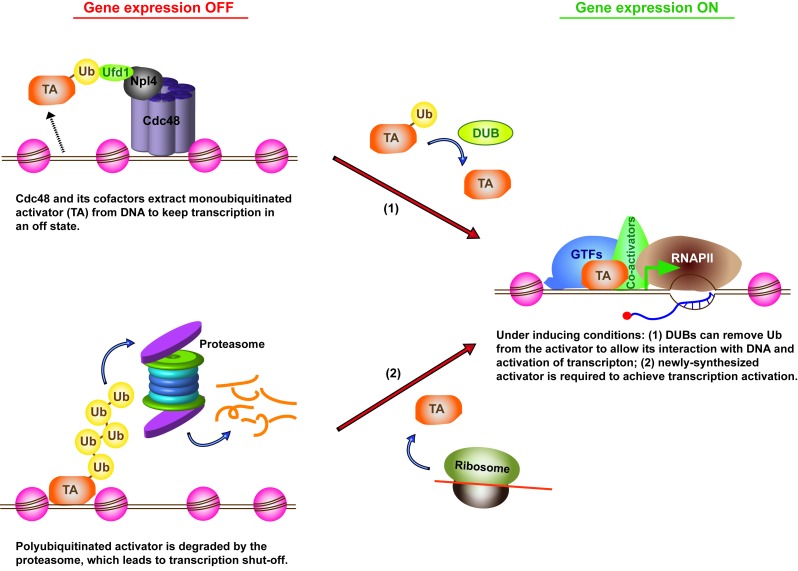
FIGURE 1: Ubiquitination of transcription activators downregulates transcription through multiple routes.

By chromatin immunoprecipitation (ChIP) assays, we found that Ub-LV has low occupancy at the promoter of a reporter gene. A key experiment was to demonstrate that this defect was due to specific properties associated with Ub. It is well known that the hydrophobic patch of Ub, composed of residues L8, I44 and V70, is a commonly used surface for Ub-protein interactions. When we introduced point mutations in the hydrophobic patch, we found that these mutations abolished the inhibitory effect of Ub fusion. Ub(L8A)-LV behaves like LV, which potently activates transcription and has a short half-life. Thus, one or more Ub-interacting proteins is responsible for preventing Ub-LV from binding to DNA. Furthermore, these results indicated that deubiquitination of the activator is required for transcription. Thus, DUBs may play a crucial role in determining the residence time of an activator at the promoter in order to finely tune the transcriptional response.

## Cdc48 extracts the monoubiquitinated activator from DNA 

Cdc48 is a hexameric AAA+ ATPase that has been termed an "Ub-dependent segregase". Previous studies from multiple laboratories had shown that Cdc48 can extract a number of ubiquitinated substrates from chromatin, such as yeast transcription repressor α2, RNA Polymerase II, and replication licensing factor Cdt1. In most cases, Cdc48 and its cofactors remove polyubiquitinated proteins from chromatin to facilitate their subsequent degradation by the proteasome. We speculated that Cdc48 also can interact with a monoUb signal and that Cdc48 was responsible for removing Ub-LV from DNA. By using temperature-sensitive alleles of Cdc48 or its cofactors Ufd1 and Npl4, we demonstrated that inactivation of Cdc48, Ufd1 or Npl4 is sufficient to restore high promoter occupancy by Ub-LV and leads to robust transcription activation. Based on the properties of Cdc48, we envision that Cdc48 functions in this case by transiently unfolding LexA, thereby causing it to dissociate from DNA and prevent transcription.

Our ChIP data demonstrated that Cdc48 was recruited to the promoter of the reporter gene in the presence of Ub-LV or Ub-LexA (without the VP16 activation domain), but not by Ub(L8A)-LV, which contains a point mutation in the hydrophobic patch. It is surprising that a single Ub attached to the activator was sufficient to recruit Cdc48 and elicit its segregase activity. After all, monoUb by itself is not a very distinctive signal *in vivo*, and competition by the large pool of unconjugated Ub in the cell is expected to weaken the interaction. We also observed substantial amounts of Ub-LV that are not bound to chromatin. These are all potential competitors for Cdc48:Ufd1:Npl4 complexes. Thus, chromatin-bound Ub-LV likely contains additional signals that contribute to recruitment of Cdc48. Through experiments that are not detailed here, we ruled out some of the usual suspects, such as SUMO modifications or transcription coactivators that may bind to VP16. We speculate that Cdc48 or its cofactor(s) has intrinsic affinity for chromatin itself. Either naked DNA, which is commonly found at promoter regions in yeast, or nucleosomes might be additional targeting signals. Low-affinity, non-specific interactions with chromatin combined with specific interactions with monoUb could target Cdc48 efficiently to promoter-bound Ub-LV. However, we cannot rule out that other components of the transcription machinery may also contribute to Cdc48 recruitment. An increasing number of proteins have been found to interact with Cdc48/p97/VCP, and a VCP-interacting motif (VIM) was recently identified in multiple Cdc48-interacting proteins. The yeast genome encodes dozens of proteins that have a consensus VIM, some of which are known players in the transcription process.

## Mono- and oligoubiquitination of yeast Met4 and mammalian R-Smads

Based on the knowledge gained from studying LexA-VP16, we sought to identify native transcription factors that are regulated similarly by monoubiquitination. Previous work from the Kaiser lab had shown that yeast transcription activator Met4 is modified by a short oligoUb chain and that this modification does not lead to protein turnover. Met4 is the master regulator of sulfur metabolism. In the absence of methionine, Met4 activates multiple genes (named *MET* genes) involved in the synthesis of sulfur-containing metabolites; however, under non-inducing conditions, cell proliferation requires inactivation of Met4, which is achieved through oligoubiquitination by the E3 ligase SCF^Met30^. Interestingly, Met4 ubiquitination without Met4 degradation is sufficient to turn off *MET* gene expression. We hypothesized that Cdc48 may play a role in maintaining the *MET* genes in a repressed state under non-inducing conditions. By measuring promoter occupancy by Met4 and monitoring transcript levels of several Met4 target genes, including *MET17*, *MET3* and *CYS3*, we showed that inactivation of Cdc48 leads to increased promoter occupancy by Met4 and partial de-repression of the *MET *genes. Importantly, in yeast strains where Met4 is not ubiquitinated, Cdc48 inactivation does not affect *MET* gene expression. These results strongly suggest that, analogous to the Ub-LV system, Cdc48 prevents ubiquitinated Met4 from stably binding to promoter DNA, thereby inhibiting transcription activation.

It was recently reported that in human cells receptor-activated Smads (R-Smads), including Smad2 and Smad3, undergo mono- and oligoubiquitination in the nucleus. This modification attenuates transforming growth factor β (TGF-β) signaling without promoting R-Smads proteolysis. We took advantage of an inhibitor of mammalian Cdc48 (i.e., p97), called DBeQ, to investigate possible involvement of p97 in R-Smads-dependent transcription. Indeed, we found that DBeQ treatment increased R-Smads-mediated transcription as well as activator promoter occupancy. These effects were observed both in the absence or presence of TGF-β, although they are more pronounced in the absence of the signaling ligand. Thus, Cdc48/p97 consistently plays a major role in maintaining target genes in repressed states through monoubiquitinated activators.

## A non-proteolytic function of Ub in transcription repression

Given the dynamic nature of Ub modification, it is often difficult to assess the direct effect of ubiquitination on transcription activators. Without specific antibodies that differentiate the ubiquitinated from non-ubiquitinated pool of activators, determining which pool is chromatin-bound and responsible for driving transcription has generally not been possible. The Ub-LV fusion we employed allowed us to bypass these obstacles. It is satisfying that the mechanism revealed from the study of the artificial activator Ub-LV applies as well to native transcription activators such as Met4 and R-Smads. We expect that additional activators will be found to be regulated by a similar mechanism in the near future.

One remaining question is why these systems employ a non-proteolytic route to achieve transcription downregulation. An obvious possibility is that mono- or oligoubiquitination is readily reversible: rapid induction of transcription by deubiquitination of the activator would not require new protein synthesis. In the case of the yeast *MET* gene network, this mode of regulation could be essential, as new protein synthesis can be limiting in an environment where methionine levels are low. Currently, the DUB that deubiquitinates Met4 upon methionine depletion has not been identified. In the case of R-Smads, Usp15 has been found to be necessary for transcription activation under inducing conditions. In these cases, how the DUBs are regulated in response to changing environments are interesting topics for future studies.

